# Immune response in hamsters immunised with a recombinant fragment of LigA from *Leptospira interrogans*, associated with carrier molecules

**DOI:** 10.1590/0074-02760160214

**Published:** 2016-10-13

**Authors:** Thaís L Oliveira, Kátia L Bacelo, Rodrigo A Schuch, Fabiana K Seixas, Tiago Collares, Oscar ED Rodrigues, Josimar Vargas, Rafaella O do Nascimento, Odir A Dellagostin, Daiane D Hartwig

**Affiliations:** 1 Universidade Federal de Pelotas, Centro de Desenvolvimento Tecnológico, Programa de Pós-Graduação em Biotecnologia, Núcleo de Biotecnologia, Pelotas, RS, Brasil; 2 Universidade Federal de Pelotas, Instituto de Biologia, Departamento de Microbiologia e Parasitologia, Pelotas, RS, Brasil; 3 Universidade Federal de Santa Maria, Departamento de Química, Santa Maria, RS, Brasil; 4 Montreal University, Chemistry Department, Montreal, QC, Canada

**Keywords:** Leptospira, LigANI, carrier molecules

## Abstract

Immunisation with the C-terminal region of leptospiral immunoglobulin-like A protein (LigANI) has shown promising results against leptospirosis. We evaluated the humoral immune response and protection induced by LigANI associated with carboxyl multi-walled carbon nanotubes (COOH-MWCNTs), CpG oligodeoxynucleotides (CpG ODNs), or Alhydrogel. Animals immunised with CpG ODNs were unable to develop a humoral immune response, whereas immunisation with LigANI and COOH-MWCNTs produced a high level of IgG antibodies, similar to that with LigANI and Alhydrogel, but it was not protective. The use of carbon nanotubes as an adjuvant in subunit vaccines against leptospirosis is a novel approach for improving specific IgG production.

Pathogenic *Leptospira* are the causative agents of leptospirosis, a zoonotic disease affecting over one million humans cases each year (Costa et al. 2015). The drawbacks of available bacterin vaccines against leptospirosis, including their side effects, short-term immunity, and serovar-restricted protection, justify the development of new prevention strategies (Dellagostin et al. 2011). Subunit recombinant vaccines consist of purified antigens and represent a possible intervention against leptospirosis. However, these vaccines lack intrinsic pathogen-associated molecular patterns and are, therefore, weakly immunogenic, thereby requiring addition of adjuvants to appropriately activate the immune system (Burnette 1991).

The non-identical carboxy-terminus portion of LigA protein (LigANI) is a vaccine candidate whose immunoprotective potential has been demonstrated in several studies. As a subunit vaccine, LigANI has been previously evaluated with Freund’s complete adjuvant (Silva et al. 2007, Coutinho et al. 2011), aluminium hydroxide (Palaniappan et al. 2006, Hartwig et al. 2014), xanthan (Bacelo et al. 2014), poly (lactide-co-glycolic acid), and liposomes (Faisal et al. 2009) as adjuvants, but not with inorganic nanomaterials.

In the past decade, inorganic nanomaterials, such as nanocrystals, nanowires, and nanotubes, have received increasing attention for their potential biomedical applications in drug design (Prato et al. 2008), drug delivery (Bhirde et al. 2009, Lodhi et al. 2013), tumour therapy (Thakare et al. 2010), tissue engineering (Zanello et al. 2006), vaccine vehicles (Yandar et al. 2008), and DNA recognition (Tu et al. 2009). Carbon nanotubes (CNTs) can be single-walled, double-walled, or multi-walled (MWCNTs) depending on the production process. Generally, CNTs are not easy to process due to their insolubility in many solvents. However, the sidewall of CNTs presents an excellent platform for chemical functionalisation, which allows overcoming the problem of processability (Niyogi et al. 2002, Pantarotto et al. 2003). For instance, MWCNTs can be functionalised with carboxyl groups to improve their dispersion in water (Zhao et al. 2010).

The demonstration that bacterial DNA, and not vertebrate DNA, has a direct immunostimulatory effect on immune cells led to the identification of the CpG class of adjuvants (Krieg et al. 1995). Preclinical studies indicate that CpG oligodeoxynucleotides (CpG ODNs) improve the efficacy of vaccines in infectious disease control (Bode et al. 2011). Here, we evaluated the IgG antibody response and protective effect induced by recombinant LigANI (rLigANI) from *Leptospira interrogans* serovar Copenhageni strain Fiocruz L1-130 in association with three adjuvants: carboxyl MWCNTs (COOH-MWCNTs), CpG ODNs, and Alhydrogel. Alhydrogel, which was included as an adjuvant in our study, is an aluminium hydroxide suspension and is a well-known standard for its ability to induce a strong humoral response (Petrovsky & Aguilar 2004). Moreover, Alhydrogel is regularly accepted for human use and is commonly evaluated in experimental leptospirosis vaccines; however, several studies have shown that IgG response to Alhydrogel is not enough to achieve protection (Adler 2015), justifying the evaluation of novel carriers or immunostimulatory molecules.

LigANI protein was expressed and purified as described by Silva et al. (2007). MWCNTs obtained from Sigma (USA) were carboxylated as described by Stefani et al. (2011). The oxidation and characterisation of COOH-MWCNTs were carried out using X-ray photoelectron spectroscopy and Raman spectroscopy. The cytotoxic effect of carbon nanotubes on Chinese hamster ovary cells was determined by measuring the reduction of soluble 3-(4,5-dimethylthiazol-2-yl)-2,5-diphenyltetrazolium bromide (MTT) to formazan, as described previously (Chiou et al. 2009), in three independent experiments. Briefly, cells were seeded in a 96-well plate at a density of 2 × 10^4^ cells/well and grown for 24 h at 37ºC in a 5% CO_2_ atmosphere. Then, the cells were treated with 2.5, 5, 10, 15, 25, and 50 µg/mL carbon nanotubes for 48 h. At the end of the treatments, 0.5 mg/mL of MTT (Sigma) was added and cells were incubated for 3 h at 37ºC. The absorbance was measured at 492 nm and the inhibition rate was determined as follows: (1 - Abs_treated cells_/Abs_control cells_) × 100%.

For vaccine formulation, an aqueous solution of COOH-MWCNTs (0.25 mg/mL) was added to reach a final concentration of 15 µg/mL (Zeinali et al. 2009). To evaluate the adjuvant activity of CpG ODNs, 10 µg of fully phosphotioated CpG ODNs (25 bp in length; 5′-TCGTCGTCGTTCGAACGACGTTGAT-3′; Alpha DNA, Montreal, Canada) was added to the vaccine formulation. Female five-six week-old Golden Syrian hamsters (*Mesocricetus auratus*) were allocated to nine groups of six animals each and were administered the following: (1) 15% Alhydrogel (InvivoGen, USA) in phosphate-buffered saline (PBS) (Alhydrogel-PBS); (2) rLigANI in PBS (rLigANI-PBS); (3) rLigANI in 15% Alhydrogel (rLigANI-Alhydrogel); (4) CpG in PBS (CpG-PBS); (5) rLigANI and CpG (rLigANI-CpG); (6) COOH-MWCNTs in PBS (COOH-MWCNTs-PBS); (7) rLigANI and COOH-MWCNTs in PBS (rLigANI-COOH-MWCNTs); (8) rLigANI, CpG, and COOH-MWCNTs in PBS (rLigANI-CpG-COOH-MWCNTs); and (9) a bacterin vaccine consisting of 1 × 10^9^ heat-killed whole-cells of *L. interrogans*, produced as previously described (Seixas et al. 2007). Two independent experiments were performed. The recombinant protein dose used for immunisation was 50 µg, which was able to induce protective immunity in another study developed by our group (Bacelo et al. 2014). The hamsters were immunised subcutaneously on day 0 and boosted on day 14. Blood was collected on days 0, 14, and 28. Sera were stored at -20ºC. After 28 days of first immunisation, the hamsters were challenged with an intraperitoneal inoculum of 1.3 × 10^3^ leptospires of *L. interrogans* serovar Copenhageni strain Fiocruz L1-130, equivalent to 5 × LD_50_. Hamsters were monitored daily and euthanised when clinical signs, such as prostration, ruffled fur, and weight loss of ≥ 10% of the animal’s maximum weight, indicating terminal disease appeared. Surviving hamsters were euthanised on day 36 post-challenge. All animal experiments were approved by the Ethics Committee in Animal Experimentation, Federal University of Pelotas, Brazil (Permit Number: 7777).

Serum IgG levels were subsequently evaluated through an enzyme linked immunosorbent assay (ELISA), using 200 ng of rLigANI as the capture antigen, as previously described (Seixas et al. 2007). The levels of anti-rLigANI IgG subclasses were also determined by indirect ELISA in two independent experiments. Briefly, 96-well plates were coated with 100 ng of rLigANI, were blocked with 5% non-fat dry milk, and serum was added in triplicates at a 1:100 dilution. A mouse anti-hamster primary antibody isotype IgG1, IgG2/3, or IgG3 (Rockland, USA) was added at a 1:100 dilution. Peroxidase-conjugated anti-mouse IgG antibody (Sigma) was added at a 1:6,000 dilution. All steps were performed at 37ºC for 1 h and the wells were washed three times with PBS with Tween-20 between each step. The reaction was developed by adding o-phenylenediamine dihydrochloride (Sigma-Aldrich, USA) and hydrogen peroxide and then stopped with 25 μL of 4 N H_2_SO_4_. The absorbance was measured at 492 nm. Differences among groups were statistically analysed using one-way analysis of variance with post-hoc Tukey’s HSD test. P values < 0.05 and < 0.001 were considered to be significant for serological assays and MTT assay, respectively. Fisher exact test was used to determine significant differences in mortality, whereas survival curves were compared using log-rank analysis (Mantel-Cox test) with Prism 6 software (Graphpad, USA).

Vaccination is considered the most economical and effective prophylactic measure against infectious diseases. In the field of leptospirosis, an efficient vaccine with cross-protection against the different pathogenic serovars remains a challenge (Adler 2015). LigANI protein was evaluated by several groups as a vaccine candidate, but its efficacy varied considerably (Dellagostin et al. 2011). LigANI represents the most promising antigen already evaluated as a subunit vaccine, conferring 67-100% immunoprotection to hamsters when associated with Freund’s or alum adjuvants (Silva et al. 2007, Coutinho et al. 2011, Hartwig et al. 2014). However, these vaccines were not able to induce sterilising immunity. Moreover, Freund’s adjuvant is associated with severe side effects and is not allowed for human use.

The use of CNTs as carriers for antigens and their ability to be internalised by different cell types have been reported in several studies (Gottardi & Douradinha 2013). CNTs are not immunogenic by themselves; however, they are able to stimulate the innate immune system, therefore having inherent adjuvant properties (Pescatori et al. 2013). In this work, we used MWCNTs as carriers of rLigANI, aiming to access the immune response induced by this antigen in hamsters. Owing to their small size, CNTs can spread within the organism, reaching crucial sites. Considering the concern about the cytotoxicity of CNTs (Yang et al. 2012), we evaluated different concentrations of CNTs in vitro using Chinese hamster ovary cells. No cytotoxic effect was observed. All concentrations tested presented inhibition rates statistically lower than that of the positive control treated with dimethyl sulfoxide (p < 0.001), not inhibiting more than 25% of the Chinese hamster ovary cells at the highest concentration (Fig. 1). Several studies have shown functionalisation as the key to improving biocompatibility and reducing cytotoxicity of CNTs (Orecchioni et al. 2014).

**Fig. 1:**
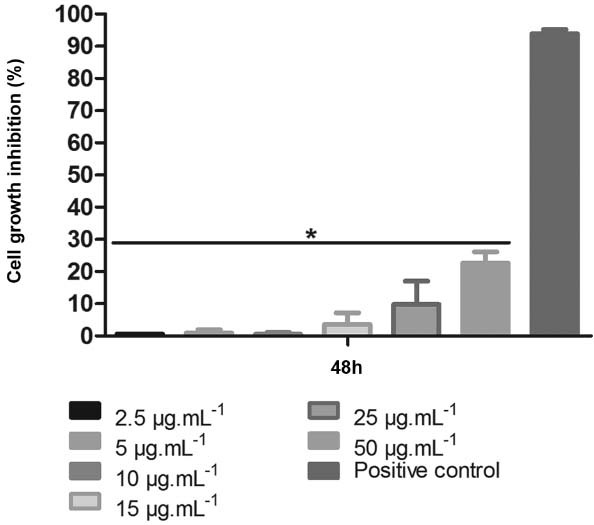
: cytotoxic effect of carbon nanotubes in CHO-K1 cells. The cytotoxicity of carbon nanotubes was assessed using MTT assay. The inhibition rate was expressed as the optical density ratio of treated cells compared to the negative control cells (only medium). The positive control cells were treated with 1% dimethyl sulfoxide. The data are expressed as mean ± standard deviation of three independent experiments. Asterisks indicate significant differences (p < 0.001) compared to the positive control.

A delivery system, such as CNT, can be used in combination with immunostimulatory adjuvants; therefore, we supplemented the vaccine formulation with CpG ODNs to determine whether the immune response could be improved (Bianco et al. 2005). The CpG motifs activate the immune system through intracellular toll-like receptor 9 (Vollmer & Krieg 2009) and cell surface receptor DEC-205 (Lahoud et al. 2012), inducing maturation of immune cells and release of cytokines by antigen presenting cells. The action of CpG DNA motifs alone is short-lived and requires administration of high and continuous doses, because both CpG and cellular membranes are negatively charged, which impairs the uptake of CpG by the cells. The ability of CNTs to enter the cells could overcome this limitation, acting as a delivery vector of CpG motifs and target antigen (Bianco et al. 2005). Hamsters immunised with rLigANI-COOH-MWCNTs or rLigANI-Alhydrogel produced a significant (p < 0.05) anti-LigANI IgG response after the first immunisation, as compared to the rLigANI-PBS group. This response was maintained at day 28, demonstrating the ability of COOH-MWCNTs to improve the humoral immune response induced by rLigANI protein (Fig. 2).

**Fig. 2:**
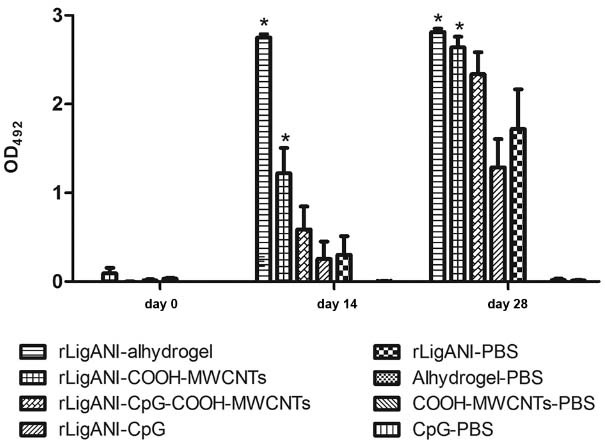
: induction of IgG antibody response in hamsters immunised with different rLigANI vaccine preparations evaluated by ELISA. Values presented are means ± standard deviation of two independent experiments. Asterisks indicate significant differences (p < 0.05) compared to rLigANI-PBS group [rLigANI- phosphate-buffered saline (PBS), recombinant non-identical carboxy-terminus portion of LigA protein in PBS].

Despite the increase in antibody levels induced by the recombinant protein associated with COOH-MWCNTs, the vaccine failed to induce a protective immune response. The survival rate in groups immunised with rLigANI-CpG and rLigANI-CpG-COOH-MWCNTs was 17% *(*p > 0.05*)*. In contrast, 67% and 100% of the animals immunised with rLigANI-Alhydrogel and bacterin survived, respectively (p < 0.05; Fig. 4). Aiming to elucidate the possible role of the type of humoral immune response in protection, we performed an indirect ELISA for isotyping of anti-rLigANI IgG subclasses (Fig. 3). Compared to rLigANI-Alhydrogel group, which conferred 67% protection, similar levels of IgG1, IgG2/3, and IgG3 were found in groups that received rLigANI and COOH-MWCNTs (p > 0.05). However, IgG1 and IgG3 responses in the rLigANI-CpG group were statistically lower than that observed in the rLigANI-Alhydrogel group (p < 0.05). Alum is an adjuvant whose mechanisms of action are well known. Here, we showed that the humoral immune response induced by carbon nanotubes is similar to that stimulated by alum. Thus, differences in the survival rate observed in our study are probably related to other routes of activation of the immune system, especially those triggered by pattern recognition receptors involved in innate immunity. The stimulation of such routes is still not well characterised for carbon nanotubes, and it should be better elucidated.

**Fig. 3:**
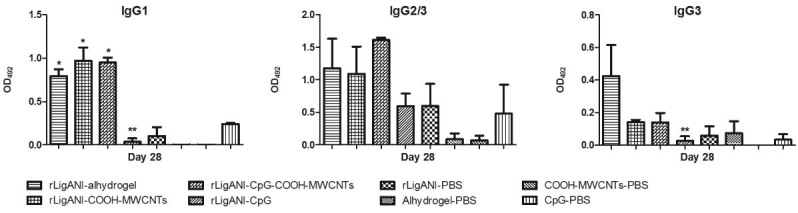
: isotyping of anti-rLigANI IgG subclasses. The data represent the difference between mean absorbance of hamster sera collected at day 28 and mean absorbance of hamster pre-immune sera at day 0. Results are expressed as mean absorbance ± standard deviation of pooled serum samples assayed in triplicates in two independent experiments. Asterisks indicate significant differences (p < 0.05) compared to rLigANI- phosphate-buffered saline (PBS) group (*) or compared to rLigANI-Alhydrogel group (**) (rLigANI-PBS, recombinant non-identical carboxy-terminus portion of LigA protein in PBS; rLigANI-Alhydrogel, rLigANI in 15% Alhydrogel).

**Fig. 4:**
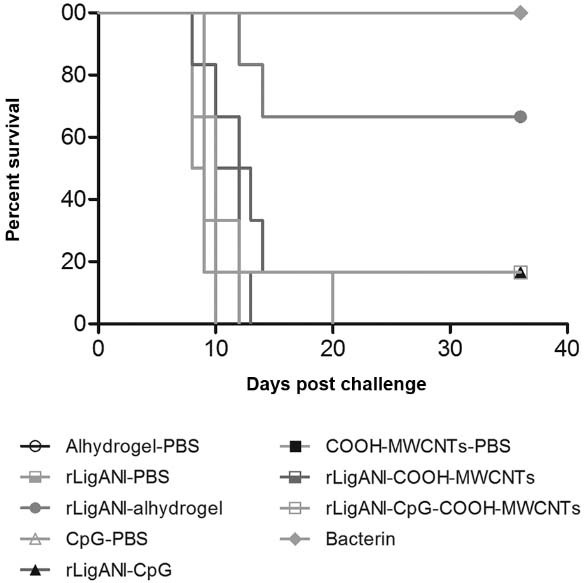
: survival of hamsters immunised with LigANI vaccines after challenge. Percentage survival conferred by rLigANI-Alhydrogel and bacterin against lethal challenge was significant (p < 0.05) in comparison to negative control group. Survival curves were compared using the Mantel-Cox test (LigANI, non-identical carboxy-terminus portion of LigA protein; rLigANI-Alhydrogel, recombinant LigANI in 15% Alhydrogel).

CNTs carrying immunogens of some pathogens have been shown to be immunogenic and protective in experimental animal models. Pantarotto et al. (2003) demonstrated the ability of CNTs in eliciting strong humoral responses and were the first to suggest their use in vaccine delivery. They linked epitopes from the foot-and-mouth disease virus to CNTs and found that, both in vitro and in vivo, this conjugation allowed the retention of correct antigen conformation and induction of a specific antibody response (Pantarotto et al. 2003). Zeinali et al. (2009) tested single-walled carbon nanotubes (SWNTs) coated with tuberculin purified protein derivative (PPD) derived from *Mycobacterium tuberculosis* in mice, and the immune response induced by immunisation with PPD-SWNT was comparable to that elicited by *M. bovis* Bacillus Calmette-Guérin (BCG); however, protection against challenge was not assessed. Immunisation with a surface protein of *Anaplasma marginale*, rMSP1a, associated with MWCNTs, significantly induced high levels of anti-MSP1a IgG (Silvestre et al. 2014). Recently, the association of halloysite and COOH-MWCNTs with LipL32 antigen from *L. interrogans* was shown to enhance IgG response at higher levels than that obtained through Alhydrogel; however, none of the animals were protected against challenge (Hartwig et al. 2015).

The results observed in the studies described above corroborate those observed in our study and show that CNTs enhance the humoral immune response. Nevertheless, the exact mechanisms by which CNTs enhance this response remain unclear. The ability of CNTs for multiple, prolonged antigen presentation, and to cross membranes, seem to be involved in their adjuvant activity (Dumortier 2013, Scheinberg et al. 2013). In conclusion, our findings suggest that COOH-MWCNTs are an effective non-toxic delivery vehicle for carrier recombinant proteins that can induce an antibody-based response. However, for leptospirosis, the humoral immune response alone seems to be ineffective in achieving protection against infection. Further studies are required to evaluate an appropriate adjuvant or delivery system that can generate a protective and sterilising immune response against leptospirosis.
